# The Rostral Migratory Stream Plays a Key Role in Intranasal Delivery of Drugs into the CNS

**DOI:** 10.1371/journal.pone.0018711

**Published:** 2011-04-13

**Authors:** Robert A. Scranton, Lauren Fletcher, Shane Sprague, David F. Jimenez, Murat Digicaylioglu

**Affiliations:** University of Texas Health Science Center San Antonio, San Antonio, Texas, United States of America; City of Hope National Medical Center and Beckman Research Institute, United States of America

## Abstract

**Background:**

The blood brain barrier (BBB) is impermeable to most drugs, impeding the establishment of novel neuroprotective therapies and strategies for many neurological diseases. Intranasal administration offers an alternative path for efficient drug delivery into the CNS. So far, the anatomical structures discussed to be involved in the transport of intranasally administered drugs into the CNS include the trigeminal nerve, olfactory nerve and the rostral migratory stream (RMS), but the relative contributions are debated.

**Methods and Findings:**

In the present study we demonstrate that surgical transection, and the resulting structural disruption of the RMS, in mice effectively obstructs the uptake of intranasally administered radioligands into the CNS. Furthermore, using a fluorescent cell tracer, we demonstrate that intranasal administration in mice allows agents to be distributed throughout the entire brain, including olfactory bulb, hippocampus, cortex and cerebellum.

**Conclusions:**

This study provides evidence of the vital role the RMS has in the CNS delivery of intranasally administered agents. The identification of the RMS as the major access path for intranasally administered drugs into the CNS may contribute to the development of treatments that are tailored for efficient transport within this structure. Research into the RMS needs to continue to elucidate its limitations, capabilities, mechanisms of transport and potential hazards before we are able to advance this technique into human research.

## Introduction

The treatment of acute or chronic neuropathological diseases with drugs poses a unique challenge to medical science due to the difficulties associated with crossing the blood brain barrier (BBB). This is the principal impediment for systemically administered drugs attempting to reach a target within the brain. Tight junctions formed by occludins, claudins and other adhesion molecules effectively block most molecules greater than 600 Daltons and limit paracellular penetration of the BBB [1]. A low Ionic strength buffer with high electrostatic potential has also been found to be important when attempting to increase BBB penetration [2]. Other possible routes across the BBB include: transcellular uptake through at least two lipid bilayers via endocytosis, carrier-mediated transport through the use of specific receptors, or peptide-mediated transport. Attempts have been made to increase BBB penetration by increasing the systemic dose; however, the concentrations necessary to penetrate this barrier and exert an effect in the central nervous system (CNS) can have deleterious systemic side effects [3]. Therefore, there is interest in identifying more direct access routes into the CNS other than systemic administration. Currently available direct modalities include intraventricular and intraparenchymal administration; cost, time, inconvenience, invasiveness and lack of efficacy make these options of poor clinical utility. A more novel route is intranasal administration, which allows agents to be rapidly delivered to the CNS and avoids the negative aspects of systemic administration. Agents are thought to traverse the nasal mucosa into the CNS via the olfactory and trigeminal nerves [4]–[6]; however, details of intranasal uptake remain elusive.

Many drugs administered intranasally have been able to reach the CNS and exert a therapeutic effect in humans. Melanocortin delivered intranasally rapidly enters the CNS without systemic spread, resulting in weight reduction after treatment for 6 weeks [7], [8]. Intranasal insulin has been studied for the treatment of diabetes, and more successfully so, in Alzheimer disease with patients showing improvements in attention, memory and cognitive function [9], [10]. Losartan administered intranasally in a mouse model of Alzheimer disease resulted in a decrease in plaque surface area and inflammatory mediators [11]. Intranasal versus intravenous naloxone was compared in a human randomized controlled trial that showed both administration routes produced equivalent responses in patients [12]. Our lab and others have shown that both erythropoietin (EPO) and Insulin-Like Growth Factor I (IGF-I), when given intranasally, can enter the CNS in high concentrations in rodent models [5], [13]–[16]. In addition, intranasally administered EPO and IGF-I rapidly accumulates in the brain at significantly higher levels than after intravenous, intraperitoneal or subcutaneous administration [14], [17], resulting in a reduction of stroke volume and improvement in behavioral function in mice. Intranasal administration of PEGylated transforming growth factor alpha was also studied in a rodent stroke model that resulted in behavioral improvements and increased proliferation of neuroprogenitor cells [18]. Kang et al. (2010) also demonstrated neuroprotection with a murine model of human immunodeficiency virus-associated neurocognitive disorders (HAND), finding that intranasal EPO + IGF-I act synergistically to activate signaling pathways which decrease the hyperphosphorylation of tau protein [19]. Tau protein hyperphosphorylation has been found in brains of both humans with HAND, and the murine model, to be associated with neuronal damage and loss.

The transport pathway taken by intranasally administered drugs into the various parts of the CNS is not completely understood. The Rostral Migratory Stream (RMS), which connects the olfactory bulb to the periventricular regions and is well described in rodents [20], [21], is potentially the missing structural link for the transport of intranasally delivered drugs into the CNS. Altman et al. (1965) observed dividing cells and neurons in the brain following injury; this led to further investigation of these cells and their destination in the non-pathologic state and the discovery of the RMS [22]–[24]. Recently, the location, route, structure, and potential function of the RMS have been further characterized in multiple animal models. The inferiolateral wall of the lateral ventricles in the mammalian brain forms the subventricular zone (SVZ). Luskin et al. (1993) observed neuroprogenitor cells arising in the SVZ appearing as interneurons in the glomerular and periglomerular layers of the olfactory bulb (OB) and hypothesized that there exists a route connecting the two areas [25]. This was designated as the RMS, a path several millimeters in length extending from the SVZ to OB. It is composed of stationary glial cells and neuronal precursors that move in chains and splay out in a radial pattern upon reaching the OB [20], [21]. It has been reported that the migration of neuroblasts coincides with a scaffold of blood vessels and astrocytes, which are in particularly high density in the RMS [26]. Dividing cells do lie in close proximity to blood vessels (BV) in the early embryonic stages, but the association is looser in postnatal stages, a possible indication that the BV scaffolding doesn't play a major role in migration; however, trophic factors could be trapped by the blood vessels providing a migratory signal [27]. Interestingly, compounds introduced into the nasal cavity of rats and mice appear in large concentrations in the OB and olfactory nerve, whereas other routes of administration do not have the same ability to penetrate the BBB [14], [28], [29]. Cells were also successfully introduced into the nasal cavity of rats and mice and found to transport into both the olfactory bulb, with “close association” to the RMS, as well as to other parts of the cerebrum via circulation through the cerebrospinal fluid [30]. In a rat Parkinson model, intranasally delivered mesenchymal stem cells were found to be widely distributed at four hours with multiple beneficial effects including reduction of inflammatory cytokines and higher levels of both thymidine hydroxylase and dopamine [31].

Many of the methods used to define the RMS in rodents cannot be applied to human research. Literature points to the presence of a human RMS, but the function at various stages of development remains to be clarified [32]. Analysis of human fetal brains has shown a ventral extension of the anterior horn of the lateral ventricles that is analogous to the rodent RMS [33]. Additionally, the distribution of doublecortin positive cells in human fetal brains indicates similarities in rodent and human neuroblast migration [33].

Most attention has focused on the caudal to rostral (anterograde) migration of neuroblasts within the RMS. Data shows that intranasal administration of small peptides such IGF-I and EPO produce high CNS concentrations in as short as twenty minutes [14], suggesting the existence of a retrograde (rostral to caudal) pathway. Little is known about a retrograde pathway or mechanism to explain this transport of peptides and cells in comparison to the better defined anterograde pathway.

In the present study, we used a fluorescent tracer, CellTracker Green BODIPY, to determine whether intranasal administration provides a sufficient pathway for delivering drugs to the brain. We found that administering a fluorescent tracer into the nasal cavity of mice produces high levels of fluorescence throughout multiple regions of the brain, including the hippocampus, cortex and cerebellum. Furthermore, in order to determine the role of the RMS in the uptake of peptides into the brain, we used radiolabeled ^125^I-EPO and ^125^I-Calcitonin. In a normal mouse, we found significant quantities of both ^125^I-cytokines in the brain 20 min after intranasal administration; however, surgical transection of the RMS abolished uptake into the brain. Therefore, we hypothesize that the intranasal pathway could provide a simple, rapid, and non-invasive means of delivering peptides into the brain via the RMS. The intranasal/RMS pathway could be applied to the treatment of many conditions including stroke, traumatic brain injury and neurodegenerative diseases.

## Materials and Methods

### Ethics Statement

All animals were housed in a supervised facility within the UT Health Science Center in compliance with the Care and Use of Laboratory Animals (Department of Health and Human Services), the provisions of the Animal Welfare Act (U.S.D.A.) and all applicable federal and state laws and regulations. The University of Texas at San Antonio Animal Care and Use Committee approved all animal care and use for this study (Approval ID: 07071).

### Animals

Male C57Bl/6 mice, average weight of 22 g, were housed in groups of 4 in a room with a 12 hour light/dark cycle with ad libitum access to food and water. Animals used for the fluorescence portion of this study were separated into two cohorts of three mice, each cohort either received treatment with a fluorescent tracer or the vehicle, phosphate buffered saline (PBS), as a control to rule out autofluorescence. Animals in the radiolabeled portion of the study were divided into cohorts of 18 who either received ^125^I-EPO or ^125^I-Calcitonin. In each of these cohorts animals were further divided into those who had surgical transaction of the RMS (n = 9) and the sham (control) group (n = 9). Three mice in each surgical group were euthanized at 20, 60, and 120 minutes.

### Preparation of Fluorescent Tracers and Iodinated Peptides

CellTracker Green BODIPY (CTG, Invitrogen, Catalog #C2102) is a low molecular weight compound of 296 Daltons that crosses cell membranes and is endogenously fluorescent. CTG contains a thiol reactive portion, which undergoes a glutathione S-transferase-mediated reaction within the cytoplasm of the cell, making it impermeable to cell membranes, thereby trapping the fluorescent tracer within the cell. Therefore, CTG provides a novel tool to determine the possible distribution pattern of an intranasally applied agent in the brain. CTG was solubilized in anhydrous dimethylsulfoxide (DMSO, Sigma-Aldrich, Catalog #276855) to a concentration of 10 mM, and then diluted to a final working concentration of 50 or 25 µM using phosphate buffered saline (PBS). ^125^I-EPO (3-[^125^I] iodotyrosyl-Erythropoietin, human recombinant [high specific activity], GE Healthcare, 2500 Ci/mmol) and Calcitonin (3-[^125^I]iodotyrosyl^2^ human calcitonin, Perkin Elmer, NEX423010UC, 2200 Ci/mmol) were dissolved separately in vehicle (10 mM sodium succinate buffer containing 140 mM NaCl, pH 6.2).

### Intranasal Delivery

Mice were placed supine on a thermo regulated surgical table that maintained body temperature at 37°C. The table was elevated to 30 degrees reverse Trendelenburg and anesthesia was induced by isoflurane (1.5–2%, 30% O_2_) delivered via facemask. Then, 6 µl of CTG, ^125^I-EPO, ^125^I-Calcitonin or vehicle was delivered to the olfactory tissue in the nasal cavity by alternate and slow administration into both nares over a period of ten minutes using a microsyringe. Mice remained anesthetized for an additional 20 minutes in the same position. The timing and position allow the tracer of interest to be taken up via the olfactory epithelium [34], [35].

### Transection of the Rostral Migratory Stream (RMS)

**Figure 1:**
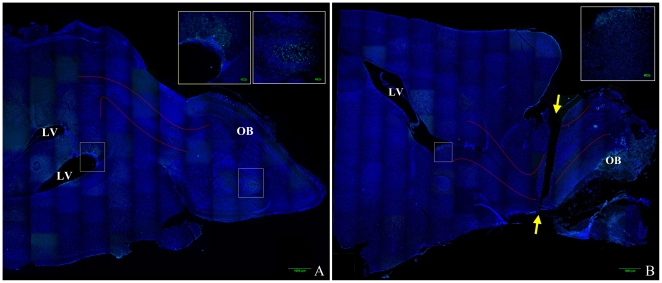
Mouse brain composite image illustrating olfactory bulb transection. Images were obtained on a Nikon confocal microscope using a 20× objective and reconstructed into a composite image using ImageJ MosaicJ. DAPI labels cell nuclei and is labeled blue, anti-BrdU labels proliferating cells and is labeled green. Figure 1A is a representative image of a control (non-transected) brain, Figure 1B is a representative image of a transected brain. Approximate location of the rostral migratory stream (RMS) is outlined between the solid red lines, lateral ventricles are labeled LV, olfactory bulb is labeled OB, and the ends of the plane of transection are labeled with yellow arrows.

Anesthesia was induced by isoflurane (1.5–2%, 30% 0_2_) and delivered via facemask to mice positioned in a stereotaxic frame and temperature maintained at 37°C with a heating blanket (Harvard Apparatus, MA). We used the surgical procedure described for bulbectomies to gain access to the olfactory bulb in anesthetized mice [36], [37]. After removing the skin over the OB area, a burr-hole was drilled into the skull covering the OB/frontal cortex, expanded to an elliptical shaped window (approx. 3×2 mm) and the location of the target tissue confirmed under a microscope at a total magnification of 100x. The tip of a scalpel blade number 11 was inserted into the window at an angle of 90° to the fronto-occipital axis of the brain, and the tissue at the distal portion of the olfactory bulb and the adjacent frontal cortex was transected (figure 1A, B). After visual confirmation of successful and complete separation of the OB, the burr-hole was closed with bone cement. To allow the complete cessation of potential bleeding and separation of the dissected tissue, intranasal ^125^I-EPO or ^125^I-Calcitonin was administered 20 min after the transection. We administered 5×10^5^ cpm of ^125^I-EPO or 5×10^5^ cpm of ^125^I-Calcitonin to each animal intranasally as described above. Sham (control) animals underwent the same surgical procedures described above, but without the transection of the RMS/OB. All animals were kept anesthetized until their designated tissue collection time (20, 60, or 120 minutes).

### Tissue Processing and Visualization

For fluorescent imaging, mice were deeply anesthetized 5 hours after intranasal administration and transcardially perfused using normal saline followed by 4% paraformaldehyde (PFA). Samples for peripheral blood smear were taken prior to perfusion. Tissue samples were harvested, transferred to a solution of 4% PFA overnight, cryoprotected in 30% sucrose in PBS for 48 hours, frozen using liquid nitrogen cooled isopentane and sectioned to 12 µm using a cryostat (Leica). Sections were incubated in 100% Acetone for ten minutes followed by three, five minute washes with PBS. Sections were then mounted on coverslips with Prolong Gold anti-fade reagent with or without DAPI (Invitrogen) and visualized using an Olympus FV-1000 confocal microscope (Olympus America Inc).

### Bromodeoxyuridine

To illustrate the location of the RMS and transection in the mouse brain, bromodeoxyuridine (BrdU, Invitrogen, 1 ml/100 g body weight, Invitrogen, Catalog #000103) was administered intraperitoneally to a mouse immediately following RMS transection, as well as a control mouse. After 24 hours brains were harvested, fixed, and cryoprotected as described above, and 35 µm sections were processed using standard immunofluorescence techniques with BrdU monoclonal antibody Alexa Fluor 488 (Invitrogen, Catalog #B35130) at 1∶100 dilution and coverslipped with Vectashield with DAPI. Images were obtained as a z-stack on a Nikon confocal microscope using a 20× objective and reconstructed into a composite image using ImageJ MosaicJ.

### Liquid Scintillation Analysis

Liquid Scintillation Analysis (LSC) measures the incorporation of ^125^I-EPO or ^125^I-Calcitonin into the brain and other tissues. Kinetic energy contained in the radioactive labeled cytokines in the tissue homogenates will be emitted as auger electrons and converted by the scintillation liquid into light energy and detected and quantified by a light sensitive detector. The total number of photons from the excited fluor molecules constitutes the scintillation. LSC was used to quantify the levels of ^125^I-EPO or ^125^I-Calcitonin after intranasal administration in control and RMS-transected mice. After administration, deeply anesthetized mice were euthanized after time periods as indicated and transcardially perfused with 10 ml of normal saline solution. This procedure is performed to remove blood from the tissues to be analyzed and to collect the blood to quantify systemic leakage of ^125^I-EPO or ^125^I-Calcitonin. After euthanasia, we collected the cerebrum without the olfactory bulb to measure ^125^I-EPO or ^125^I-Calcitonin uptake into the brain. Lung tissue was also collected to test whether the radioligands were aspirated, and a swab of the nasal cavity was performed with liquid absorbing filter paper to collect residual amounts of the radioligands in the nasal cavity. All homogenized tissue samples and nasal swabs were individually mixed with a liquid scintillation cocktail (1 ml in 50 mM Tris-HCl, pH 7.4, Microscint, Packard) to a final volume of 12 ml in a scintillation glass vial and measured, in presence of calibrators (Perkin Elmer, I-125 Pico-Calibrator, Catalog #5080125), in a scintillation counter (Topcount, Packard). Measurement controls with quenched samples (with nitromethane) and scintillation cocktail without radioactivity were run in parallel as controls. LSC measurements are expressed as the percent of radioactivity detected in the samples compared to the amount applied. All statistical analysis was performed using one-way ANOVA and a *p-value<0.01* was taken as statistically significant.

## Results

### Intranasal Administration of CellTracker Green

**Figure 2:**
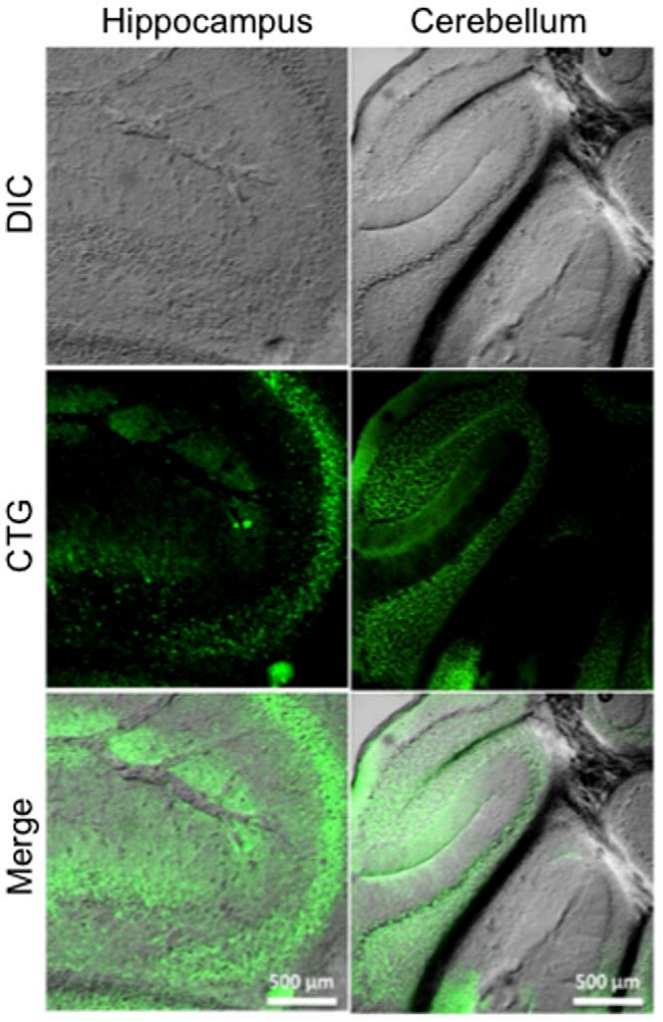
Intranasal uptake of CTG into the hippocampus and cerebellum. Anesthetized mice received intranasal administration of CTG (6 µl) or vehicle (PBS) into both nares slowly over a period of ten minutes. Mice remained anesthetized for an additional 20 minutes in a supine position to allow absorption of the tracer through the olfactory epithelium and into the olfactory blub. Five hours later, mice were euthanized and perfused with 4% PFA, and the brains sectioned into 12 µm slices. CTG positive cells are labeled green. Vehicle treated mice did not produce any signal (data not shown).

**Figure 3:**
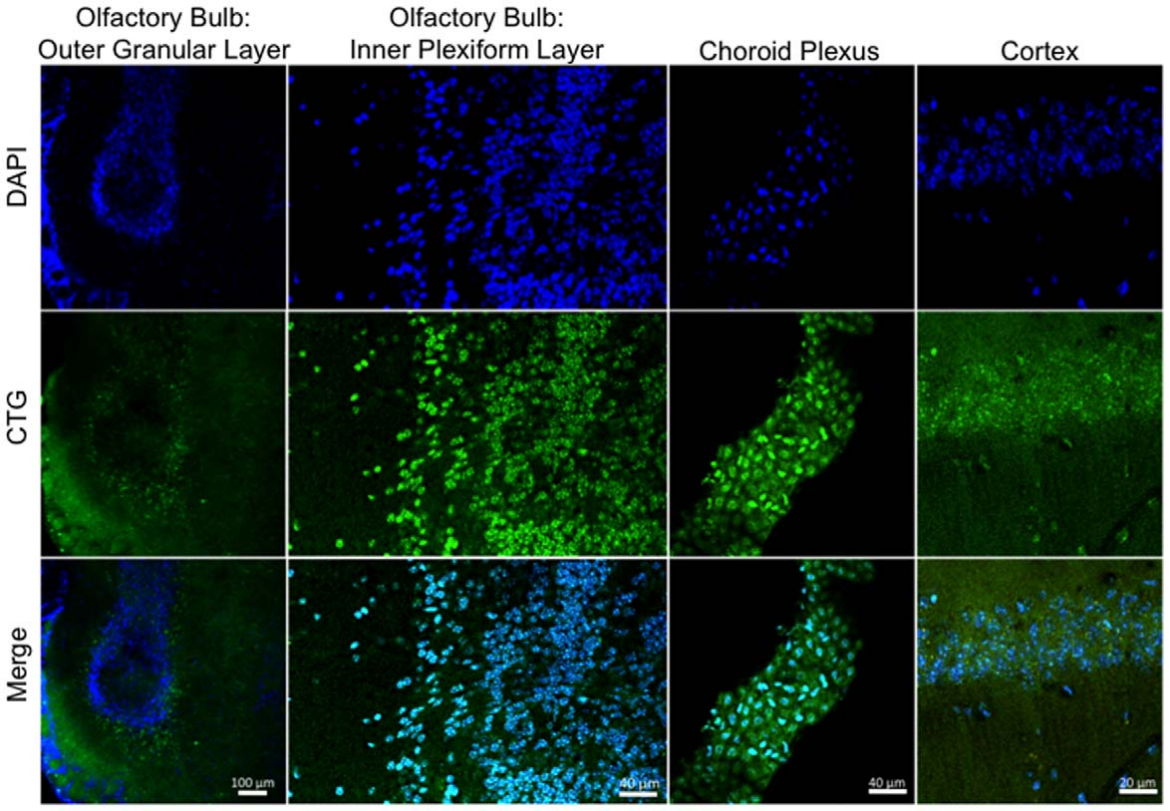
Intranasal uptake of CTG into the olfactory bulb, choroid plexus, and cortex. Anesthetized mice received intranasal administration of CTG (6 µl) or vehicle (PBS). Mice remained anesthetized for an additional 20 minutes in a supine position. Five hours later, mice were euthanized and perfused with 4% PFA, and the brains sectioned into 12 µm slices. Nuclei were counterstained with DAPI (Blue). CTG cells are labeled green. No signal was detected in the vehicle treated mice (data not shown).

**Figure 4:**
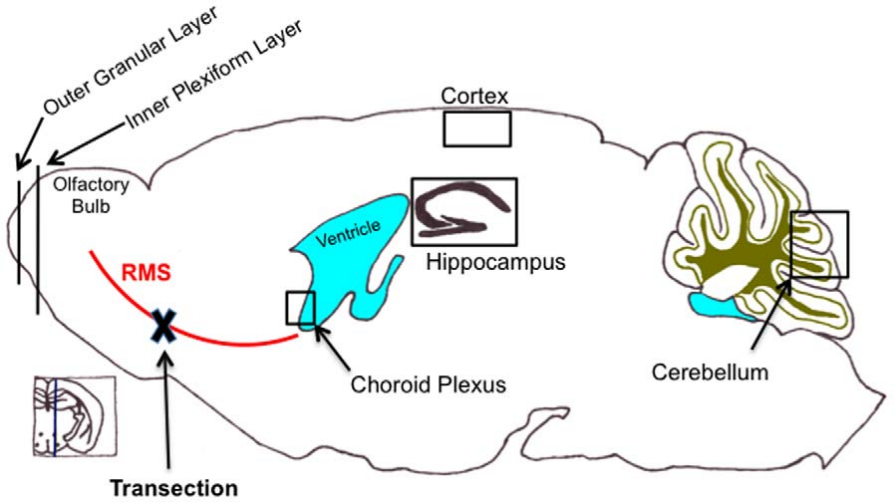
Illustration showing the location of the RMS, where it was transected, and its relation to where images were obtained for **Figures 2** and **3**.

Intranasally delivered CTG (6 µl) was detected by microscopic detection of CellTracker Green throughout the hippocampus and cerebellum 5 hours after administration (Figure 2). CTG positive cells were also detected in the olfactory bulb, cortex, and the choroid plexus (Figure 3). Figure 4 demonstrates the areas of the mouse brain that were imaged for Figures 2 and 3. CTG positive cells were not observed in any peripherial tissue sample such as kidney, lung, and liver from any subject in the CTG or control group (images not shown). Peripherial blood smears to assess for hematogenous dissemination of CTG were negative for any CTG positive cells (images not shown).

### Intranasal Administration of ^125^I-EPO & ^125^I-Calcitonin after RMS Transection

**Figure 5:**
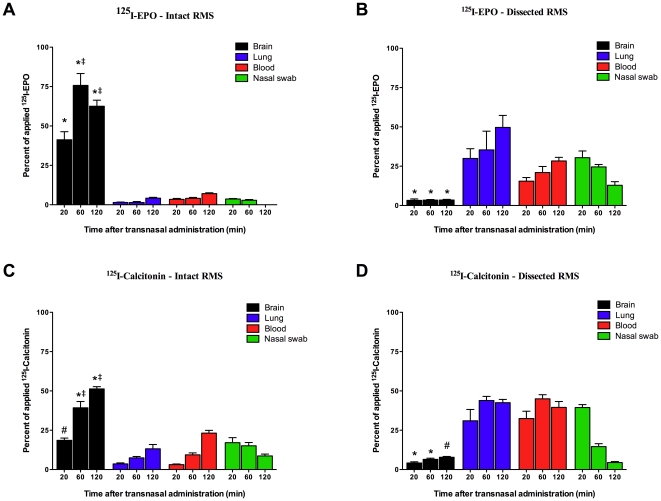
Disruption of the RMS prevents intranasal delivery of peptides. Mice were anesthetized and the RMS dissected through a small burr-hole over the olfactory bulb. ^125^I-EPO or ^125^I-Calcitonin was administered intranasally 20 minutes later. The mice were then euthanized after 20, 60, or 120 minutes, and radioactivity was measured in brain tissue, lung tissue, blood and nasal swabs using a scintillation counter (Perkin Elmer). Data is expressed as a percentage of the total amount of the applied radioligand (mean ± SD, n = 3). *p<0.01, percent of radioligand in the brain compared to lung, blood and nasal swabs at each time point. ^#^p<0.01, percent of radioligand in the brain compared to lung and blood only, ^‡^p<0.01, percent of radioligand in the brain at 60 or 120 minutes versus in the brain at 20 minutes.

The RMS in mice is located within the olfactory tract (OT) [38]. To understand its role in the transport of peptides into the brain after intranasal administration, we surgically transected the RMS, thereby disrupting the normal flow. ^125^I-EPO or ^125^I-Calcitonin was administered intranasally to RMS transected and sham (control) mice. Mice were euthanized 20, 60 and 120 minutes following administration. Additional samples from the blood, lungs, and nasal swabs were used to quantify systemic leakage from both surgical and control groups. Samples were then analyzed using liquid scintillation analysis (Figure 5). In the control group (intact RMS), 20 minutes after administration, 41.3%±5.1% of the applied intranasal ^125^I-EPO and 18.6%±1.4% of the applied intranasal ^125^I-Calcitonin was detected in the brain, without significant amounts in the peripheral homogenates including lung, blood and nasal swabs (Figure 5A, 5C). It is of interest to note that 75.8%±7.5% of the applied ^125^I-EPO is in the brain after only 60 minutes. However, in the mice with a transected RMS, only 3.3%±0.9% of the applied intranasal ^125^I-EPO and 4.2%±0.7% of the applied intranasal ^125^I-Calcitonin was detected in the brain samples with significant quantities found in peripheral sites (Figure 5B, 5D).

Systemic leakage of intranasally administered ^125^I-EPO was statistically negligible and did not significantly increase during the observation period. Nasal swabs indicated that an insignificant amount of ^125^I-EPO remains in the nasal cavity. In a similar fashion, systemic leakage of intranasally administered ^125^I-Calcitonin was not statistically significant when compared to CNS, but higher than the values for ^125^I-EPO. However, systemic leakage for both radioligands increased significantly in mice subjected to RMS transection, and high residual amounts of ^125^I-EPO and ^125^I-Calcitonin were detectable in the nasal cavity.

## Discussion

In the present study the intranasal administration of low molecular weight fluorescent probes and radioligands results in accumulation of fluorescence and radioactive signal throughout the brain, but not in peripheral tissues such as lungs and blood. However, when the RMS is surgically transected, the radiolabeled markers are found mainly in the peripheral organs, with no statistically significant quantities in the brain. The volume chosen for administration into the nasal cavity (6 µl) has been shown previously to be the maximum volume that can be applied to the olfactory tissue without subsequent systemic leakage. Therefore, the presence of the radioligands in the peripheral tissue is unlikely due to the volume administered into the nasal cavity. We hypothesize that structural disruption of the RMS prevented uptake into the CNS. This resulted in prolonged contact with the respiratory tissue allowing the radioligands to take another path, resulting in an increase in radioactive signal in the lung and blood samples. We observed an increase in mucosal volume in the nasal cavity after RMS transection. This increase in fluid volume may also contribute to the likelihood that the radioligands are taken up systemically via the circulatory system or aspirated into the lungs. This is supported by the fact that the radioactivity is initially high in the nasal swab samples, but diminishes over time. Moreover, these results are indicative of the rapid uptake by the RMS which, when intact, does not allow for the buildup of radioligands within the nasal cavity. This is further supported by our findings that ^125^I-EPO and ^125^I-Calcitonin are found in the CNS in significant quantities within twenty minutes when compared to locations outside the CNS. Therefore, our findings suggest that a structurally intact RMS is necessary for the rapid uptake of peptides into the CNS.

The trigeminal and olfactory nerves have been discussed as potential access routes from the nasal cavity into the CNS [6], [13], [39], [40]. In rodents the RMS is embedded into the olfactory nerve making RMS transection without injuring the olfactory nerve surgically impossible. Therefore, we cannot exclude the role of the olfactory nerve. However, were the olfactory nerve the main route into the CNS, a more focused accumulation of the tracer and radioligands in the projection areas of this nerve would be expected. Our previously published results from autoradiographs indicate that intranasally administered radioligands distribute throughout the brain and are not confined to or concentrated in the olfactory areas innervated by the olfactory nerve [39]. Moreover, tracers used in the present study did not show a preference for the projection areas of the olfactory nerve. These results indicate that either: a) the RMS, not the surrounding olfactory nerve is the main transport route, or b) substances transported by the olfactory nerve bypass the olfactory region of the brain. In this model the trigeminal nerve remains intact and may provide a potential uptake path into the CNS. However, the dramatic reduction of cerebral uptake of intranasally administered peptides following the transection of the RMS indicates that the trigeminal nerve may play a lesser role in CNS transport within the time frame of this study. Nevertheless, it is entirely possible that all structures named above might collectively provide an access path into the brain, but with different rates of transportation and different target areas. Therefore, subsequent experiments that involve longer administration periods than used in this study are needed.

The mechanisms of transport within the RMS are not thoroughly addressed by this study; however, the rapidity of uptake of both the fluorescent tracer and radioligands suggest a paracellular mechanism rather than cell mediated. In addition, a greater concentration of the fluorescent tracer would be expected in the more rostral cells along the RMS were the mechanism involved cell mediated or transcellular. This suggests that it is the existence, and to some degree, the structure of the RMS that is necessary, rather than the functionality; i.e. the movement of cells. This makes surgical transection of the structure a desirable method to evaluate its role. Severe reactive astrogliosis with scar formation is likely to occur in the long term as a result of the direct trauma caused by transection [41]. Astrocyte hypertrophy would only be at the beginning stages at twenty minutes, gene up-regulation such as GFAP mRNA takes an hour to be detected, and glial scar formation would take greater than six hours [42], [43]; however, reactive astrogliosis and scar formation is unlikely to have a significant effect in the short time frame of this study. The earliest time-point at which hypertrophy is detectible in rats is 24 hours after insult, reaching maximal response in 3–4 days; the human response is further delayed with detection and maximum around 4 days and 2–3 weeks, respectively [44]. Interestingly, studies using a longer time frame than this study in evaluating the movement of cells after RMS transection, demonstrated PSA-NCAM positive cells penetrating the glial scar [45].

We also cannot discount the possibility that results may be different in genetic models with a dysfunctional RMS, often resulting in olfactory bulb hypoplasia [46]–[48]. However, in these models some degree of cellular migration along the RMS is still present; more important, the architecture of the RMS remains intact. Kaneko et al. (2010) recently provided evidence suggesting that the Slit1 protein is used by new neurons to form and maintain astrocyte tunnels, as well as alter astrocyte morphology [49]. This model creates RMS dysfunction through structural alteration and the results of repeating this investigation using Slit1 deficient mice would be interesting; however, it is unclear whether the degree of RMS structural disruption in Slit1 deficient mice is comparable to RMS transection.

Numerous publications confirm the efficacy of intranasal administration for the delivery of compounds and cells to the CNS with the added benefit of minimal systemic spread [6], [11], [18], [31], [50]. Various strategies have been employed in an effort to increase uptake such as nanoparticles and PEGylation; however, the intranasal pathway is so efficient these had minimal effect or were negated by optimizing the ionic strength of the buffer used [18]. Not all studies have had a successful treatment effect despite high CNS concentrations of the compound administered [51]. This illustrates an important caveat, the intranasal pathway is not a cure-all for CNS disease, but it is a highly efficient means of delivering agents that may or may not have their own unique target and mechanism. Prior studies hypothesized the pathway taken to the CNS following intranasal administration to be a combination of the olfactory and trigeminal nerve. However, a literature search failed to produce any studies that used a physical transection of the proposed paths to demonstrate loss of transport into the CNS. This study shows that using intranasal administration, transection of the RMS decreases CNS concentrations of radio-labeled peptides by over 80%. This suggests that the RMS is the major pathway used following intranasal administration.

The intranasal pathway could provide an inexpensive, non-invasive, and effective means of gaining high concentrations of agents in the CNS without systemic side effects. This pathway could be applied to the treatment of many conditions including traumatic brain injury, stroke, and neurodegenerative disease. This study has provided evidence of the vital role the RMS has in the CNS delivery of intranasally administered agents. The identification of the RMS as the major access path for intranasally administered drugs may contribute to the development of therapeutics tailored for efficient transport within this structure. The transport capacity of the RMS is likely to be influenced by the physiochemical properties of administered substances such as molecular weight, solubility, charge and dissociation characteristics. Research into the RMS needs to continue to elucidate its limitations, capabilities, mechanisms of transport and potential hazards before we are able to advance this technique into human research.
